# Convolutional Neural Network-Based Drone Detection and Classification Using Overlaid Frequency-Modulated Continuous-Wave (FMCW) Range–Doppler Images

**DOI:** 10.3390/s24175805

**Published:** 2024-09-06

**Authors:** Seung-Kyu Han, Joo-Hyun Lee, Young-Ho Jung

**Affiliations:** 1 School of Electronics and Information Engineering, Korea Aerospace University, Goyang-si 10540, Republic of Korea; skhan980401@kau.kr; 2 Datalink 2 Team, Hanwha Systems Co., Ltd., Seongnam-si 13524, Republic of Korea; hmly7606@hanwha.com; 3 Department of Computer Engineering, Korea Aerospace University, Goyang-si 10504, Republic of Korea

**Keywords:** drone detection, FMCW radar, convolutional neural network, range–Doppler map, micro-Doppler signature (MDS), overlay

## Abstract

This paper proposes a novel drone detection method based on a convolutional neural network (CNN) utilizing range–Doppler map images from a frequency-modulated continuous-wave (FMCW) radar. The existing drone detection and identification techniques, which rely on the micro-Doppler signature (MDS), face challenges when a drone is small or located far away, leading to performance degradation due to signal attenuation and faint (MDS). In order to address these issues, this paper suggests a method where multiple time-series range–Doppler images from an FMCW radar are overlaid onto a single image and fed to a CNN. The experimental results, using actual data for three different drone sizes, show significant performance improvements in drone detection accuracy compared to conventional methods.

## 1. Introduction

As the use of drones increases, the number of accidents related to drone flights also increases. Systems for detecting drones are required to prevent such accidents in advance; however, it is not easy to capture the characteristics of drones when they are far away. Utilizing radar systems to detect drones has been an effective method for a long time; however, conventional radar systems can only determine a target’s position and velocity and have difficulty distinguishing between objects such as drones, birds, and other objects. Therefore, the micro-Doppler signature (MDS), a characteristic that occurs via a dynamic drone propeller’s rotation, is popularly utilized to analyze the features of drones and identify them as such [1,2,3,4,5,6,7,8,9,10,11].

In drone detection research using radar technology, a continuous-wave (CW) radar, with which to detect and differentiate between drones, utilizing the MDS has been studied [2]. CW radars have the advantage of simple hardware and the capability to detect moving objects as well as measure their speed; however, they have the drawbacks of being relatively susceptible to noise and unable to obtain distance information. An increasing trend exists in the research of using frequency-modulated continuous-wave (FMCW) radars to compensate for the shortcomings of CW radars.

The time–velocity diagram (TVD) is a graph-type diagram representing time on the horizontal axis and velocity on the vertical axis, and it is categorized as one type of MDS. This diagram visually illustrates the change in an object’s velocity over time, allowing for the observation of how the velocity of an object changes during a specific period. Study [3] utilized a TVD, containing the characteristics of each object, for drone detection, comparing detection performance using various classifiers, such as a support vector machine (SVM). Furthermore, study [4] classified targets using TVD images containing features of drones and birds through a CNN model. Study [5] improved drone detection accuracy by eliminating clutter components in a TVD. The study conducted by the authors of [6] obtained TVD data for drones with different numbers of propellers and differentiated between them by using a CNN model. Although a TVD proves helpful in drone detection and classification, it provides information solely on time and velocity, lacking various dynamic details such as object position, direction, and rotation.

The range–Doppler map, a type of MDS, has velocity on the horizontal axis and target distance on the vertical axis and is popularly used for drone detection and identification [7,8,9,10,11]. This image combines Doppler effects generated when radar waves sent to the target are reflected, along with distance information to the target. Through this image, information about how the target moves can be discerned. Study [7] differentiated objects using range–Doppler maps for drones, cars, and people. Moreover, ref. [7] separated the Doppler characteristics of range–Doppler maps and classified drones using a linear classifier. Study [8] utilized an FMCW radar with K-band frequencies to create range–Doppler maps and applied them in drone and object detection research. In [9], research on detecting the presence or absence of drones at long distances using range–Doppler maps was presented. In [10], drone type identification utilized the unique MDS characteristics of each drone in the range–Doppler map.

In [11], multiple sensor-fusion-based drone detection and classification methods were studied. When an FMCW radar is used, conventional range–Doppler images are input into a CNN model; however, a drawback of drone detection or identification utilizing a range–Doppler map is the degradation of the MDS due to an increase in distance, leading to compromised detection and identification performance.

This paper proposes an “image overlay” method for accurately detecting long-range drones to address this issue. This approach involves overlaying range–Doppler images generated chronologically by an FMCW radar, sequentially creating composite images. The proposed image overlay method stacks the range–Doppler data from bottom to top, storing the data for each image and then overlaying subsequent range–Doppler data on the excluded portions. In other words, the range–Doppler data over multiple images are merged into a single image. Using the overlaid range–Doppler images obtained for three different types of drones, transfer learning models based on CNNs such as AlexNet [12], SqueezeNet [13], and GoogLeNet [14] are employed to observe the effect of the proposed image overlay method on drone detection accuracy. According to performance evaluation results for a wide range of distances, a meaningful performance improvement in the proposed image overlay method is observed.

The method of overlaying time-series range–Doppler images proposed in this paper is simple compared to methods such as those of [15], which combine time-series data by weighting them using machine learning methods. However, general commercial radar systems often do not provide intermediate raw data that can be used for additional signal processing; they only provide preprocessed image data through proprietary software. This study is meaningful because it is a practical method that improves performance using only provided image data, such as range–Doppler maps. 

The structure of this paper is as follows: Section 2 introduces the characteristics of a drone MDS and prior research utilizing them. Section 3 presents the proposed image overlay method. Section 4 describes the types of equipment and experimental environment used, while Section 5 presents the deep learning networks used in the simulation and shows experimental results. Finally, Section 6 provides the conclusions derived in this paper.

## 2. Background of Research

### 2.1. Micro-Doppler Signature

When an object undergoes mechanical vibration, rotation, or significant movement, a frequency shift in the received signal, known as the Doppler frequency shift of the target, is generated. This phenomenon is referred to as an MDS. Due to the propellers rotating at a consistent frequency, drones exhibit a distinct MDS pattern. This pattern effectively identifies characteristics such as a drone’s type, size, and flight state. The Doppler spectrum for a drone with P rotors and N blades for each rotor is accurately analyzed in [16], and the final form of it can be re-expressed as follows:

(1) $$S\left(f\right)=\bar{A}\delta \left(f+\frac{2v}{\lambda }\right)+\sum_{p=1}^{P}\sum_{m=-\infty }^{\infty }{\bar{B}}_{pm}\delta \left(f+\frac{2v}{\lambda }-\frac{\omega_{p}}{2\pi }Nm\right),$$

where $\delta \left(\cdot \right)$ is the Dirac delta function, p is the index of the rotor, v is the velocity of the drone, λ is the wavelength of the signal used in the radar system, and $\omega_{p}$ represents the angular velocity of the $p_{th}$ rotor. In the Doppler spectrum, $S\left(f\right)$ is composed of a refracted signal from the main body of the drone, which has a Doppler shift of $\frac{2v}{\lambda }$, and signals with pairs of $\frac{mN\omega_{p}}{2\pi }$ frequency shifts with a reference to the center frequency of $\frac{2v}{\lambda }$. These pairs of harmonic frequencies are multiples of the chopping frequency, which can be expressed as follows:

(2) $$Chopping\;Frequency=\frac{N\omega_{p}}{2\pi }\;$$

As shown in the second part of Equation (1), the MDS spectrum generated from the blades is evenly positioned on both sides relative to the primary Doppler of the drone’s body. Additionally, as N and $\omega_{p}$ increase, the value of the chopping frequency also increases. 

In Equation (2), $\bar{A}$ and ${\bar{B}}_{pm}$ represent the reflected signal’s complex amplitude due to the drone body and the $m_{th}$ harmonic frequency of the $p_{th}$ blade, respectively. They denote the complex amplitudes received from scatterers, encompassing both magnitude and phase information, which characterize the properties of each scatterer. $\bar{A}$ varies depending on the size and shape of the drone, and ${\bar{B}}_{pm}$ depends on parameters such as the angle of incidence of the $p_{th}$ rotor, material properties affecting the scatter, and the geometric configuration of the radar system.

FMCW radars use chirp signals to measure a target’s distance and velocity. This is accomplished by computing the frequency difference between the transmitted and received signals, also known as the beat frequency. Fast Fourier transforms are performed on the beat frequency signal in both range and velocity directions to create a range–Doppler map.

**Figure 1:**
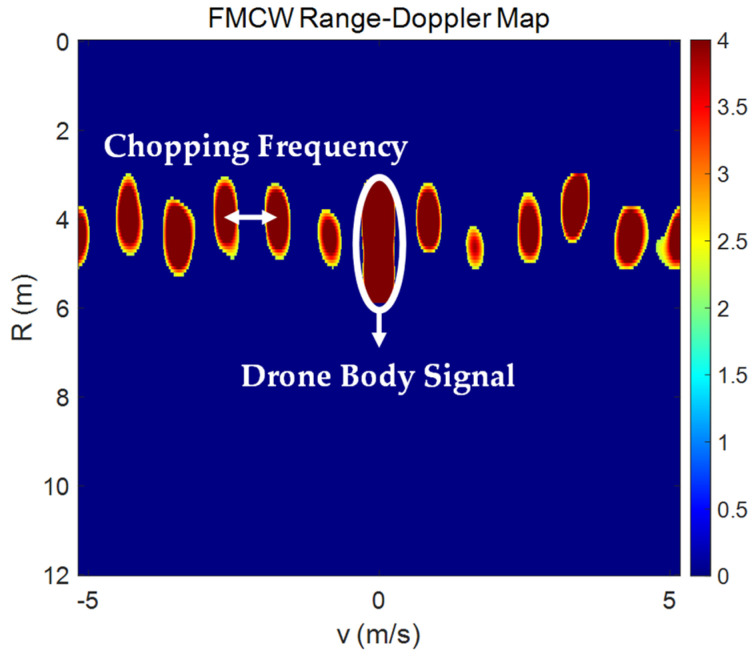
An example of a range–Doppler map for a hovering drone.

**Figure 2:**
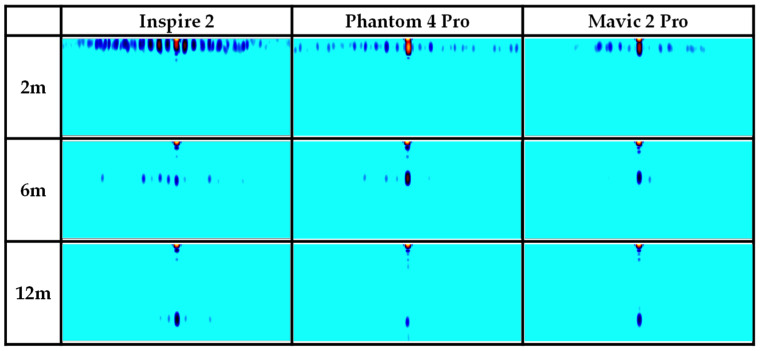
Range–Doppler map of drones at distances of 2, 6, and 12 m.

Figure 1 illustrates an example of a range–Doppler map obtained from measuring an Inspire 2 drone model using an FMCW radar at a distance of 4 m. The white circles in the image signify MDS, while the regions marked with white bidirectional arrows represent the chopping frequencies. Figure 2 lists the range–Doppler maps of three drone models for various distances. The Inspire 2 model, being the largest, shows the most distinct MDS characteristics compared to the other models; however, as the distance increases, it is observed that the MDS signals for all three models gradually weaken due to signal attenuation. This makes it challenging to detect drones at longer distances. Therefore, this paper introduces the image overlay method to enhance the clarity of attenuated MDS signals. The goal is to improve drone detection performance even at long distances.

### 2.2. Related Research Results

**Figure 3:**
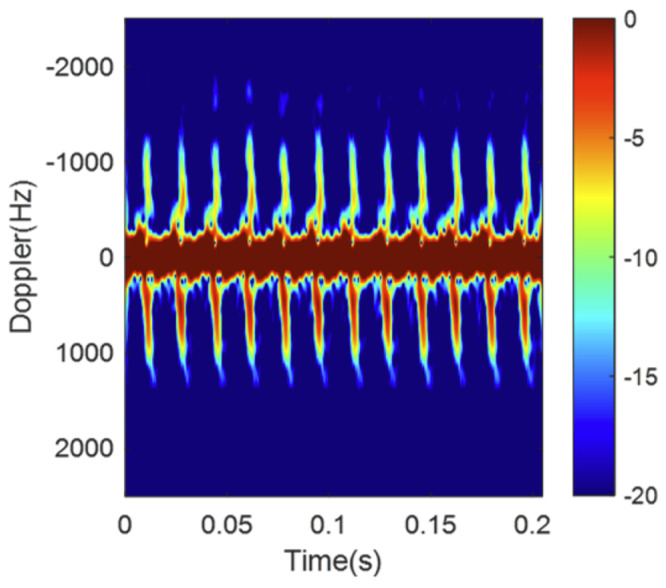
An example of a TVD image for a hovering quadcopter [2].

Table 1 presents previous research results using an FMCW radar for drone detection. All the listed studies utilized an MDS to detect drones. Study [3] differentiated drones, birds, and noise by using TVD images similar to those in Figure 3. Furthermore, studies [6,7] developed this approach to classify various drone models. In contrast, refs. [10,11] used range–Doppler map images (Figure 2) to distinguish between different drone models. Moreover, ref. [9] used these images to differentiate drones from non-drone targets such as cars or people. These studies achieved high accuracy levels by training a CNN model with range–Doppler map images. This research aims to replicate the environments described in [10,11] as closely as possible to compare the performance and highlight the effectiveness of the proposed overlaying method in this paper.

## 3. Proposed Image Overlay Method

### 3.1. Image Overlaying Process

**Figure 4:**
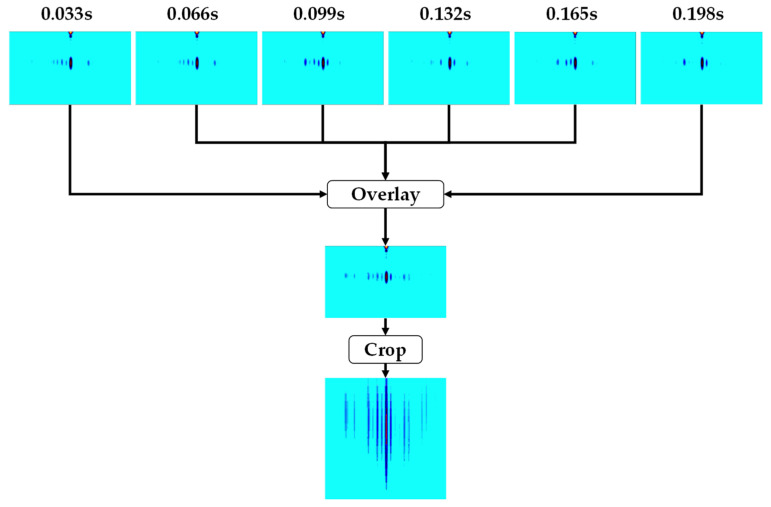
An example of an overlaying process with six images in time series.

Figure 4 illustrates the proposed image overlay method for enhancing the MDS of a range–Doppler map by overlaying multiple images. The example combines six range–Doppler images with intervals of 0.033 s, sorted in chronological order, to create a single overlaid image containing the MDS of all the images. The MDS characteristics appear clearer with an increase in the number of images; however, the number of images should be optimized due to limited image data and processing speed. Additionally, since the objective is to detect drones by using only the MDS portion of the images, the portion without an MDS is removed. When the range–Doppler image generation cycle is fixed depending on the characteristics of an FMCW radar, there is the advantage that MDS features are strengthened as the number of overlaying images increases. Still, there is a problem in that the overlaid image generation cycle becomes longer and computational complexity increases; therefore, it is necessary to optimize the number of overlaying images, which will be covered in Section 4.2.

### 3.2. Image Overlaying Algorithm

**Figure 5:**
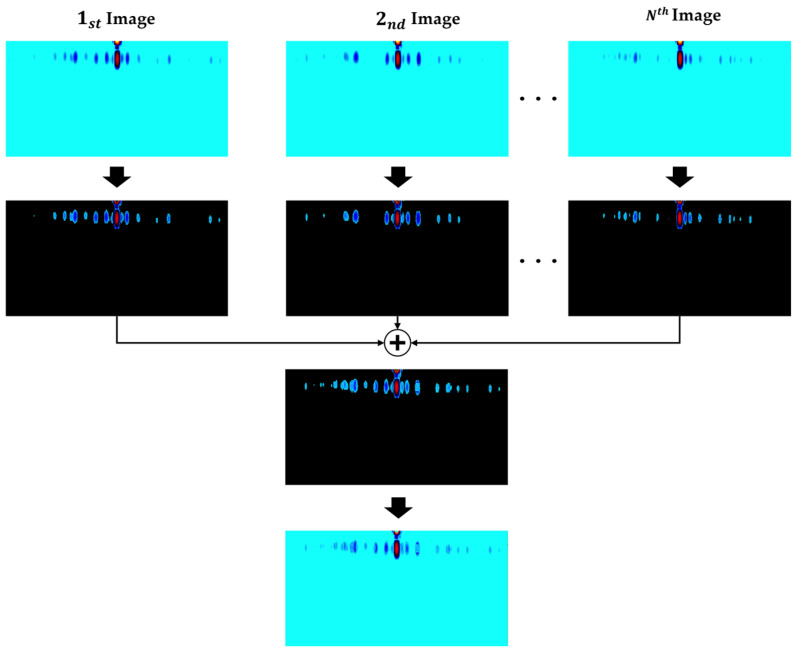
The image overlaying process for MDS enhancement.

The image overlay method, a step-by-step process, involves separating the MDS area and the background area of multiple images and then overlaying the MDS area of all the images. Figure 5 presents the algorithm for the image overlay method. When overlaying time-series images, the MDS areas of the images are separated, and their background areas are filled with a black color. After this, the images containing the separated MDS areas are merged, and the black areas are filled with the original background color of the images. This process creates a single range–Doppler map image that contains the MDSs from the time-series images. Consequently, this generates an overlaid image, which accumulates MDS information over time.

## 4. Experimental Environment

### 4.1. Experimental Equipment

**Figure 6:**
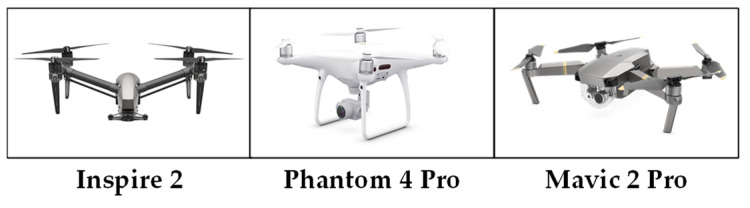
The models of the drones used in the experiment.

Experiments were carried out to evaluate the effectiveness of the proposed image overlay method in an open outdoor area without any obstacles that could prevent the radar from detecting objects other than drones. To tackle the challenge of reduced signal strength and the resulting difficulty in detecting drones as they move farther from the radar, three quadcopters of different sizes from DJI (Shenzhen, China) and a non-drone object (a corner reflector) were used as targets, as in Figure 6. The angular velocity at the end of the rotor is directly proportional to the length of the propeller, leading to an increase in the chopping frequency, as shown in Equation (2). Additionally, heavier drones’ motors have more revolutions per minute (RPM), resulting in a more distinct MDS signal. According to [17], a longer rotor length leads to a larger radar cross-section (RCS), which in turn increases the signal intensity [18]. Thus, it is expected that different drones will have unique MDSs. As shown in Table 2, the Mavic 2 Pro (DJI, Shenzhen, China) is the smallest and lightest of the three drones, while the Inspire 2 (DJI, Shenzhen, China) is the largest and heaviest. Therefore, it is predicted that the MDS of the Inspire 2 will be the most distinct and pronounced. The FMCW radar used for data acquisition in this study is the Eval-DEMORAD radar (Analog Devices, Inc., Wilmington, MA, USA) [19]. Its specifications can be found in Table 3.

### 4.2. Outdoor Test Environment and Dataset Construction

**Figure 7:**
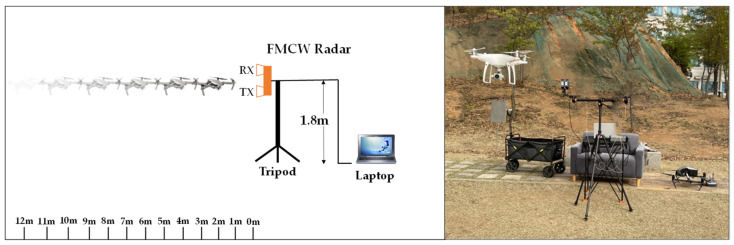
Experiment for collecting data in an outdoor environment.

The outdoor experiment setting for our experiments is illustrated in Figure 7. We mounted an FMCW radar at a height of 1.8 m. We collected data from hovering drones at distances ranging from 2 to 12 m, with 1 m intervals. The data were saved in real time on a laptop and later used for image processing. The range–Doppler map images provided by DEMORAD Radar’s software (INRAS version 2.4.0) are displayed at 30 frames per second (fps). By capturing these images at intervals of 0.033 s on a laptop, a dataset of 3600 range–Doppler map images for each distance and drone model was created. 

To determine the optimal number of overlaying images that can trade off performance and complexity, we simulated the accuracy of drone and non-drone image classification according to the number of overlaying images using an AlexNet-based machine learning model. The accuracy was measured using 1000 overlaid drone and non-drone images at a distance of 6 m to observe reasonable performance. The machine learning parameters used in the experiment are shown in Table 4.

**Figure 8:**
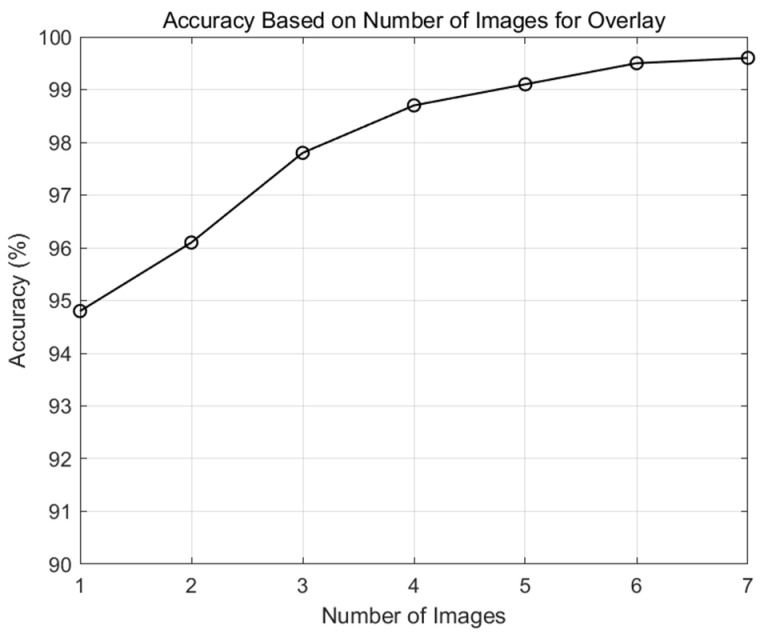
Drone classification accuracy based on the number of images for overlays.

Figure 8 shows the accuracy of drone classification as the number of overlaying images changes. The accuracy improves as more images are used for overlays, but this improvement saturates when the number of overlays exceeds six. According to the results, we set the number of overlaying images to six to balance the performance and complexity. To confirm the possibility of real-time processing, we tested the processing time for the image overlaying process. Applying an image overlaying algorithm with MATLAB 2024a using a personal computer with an i7 11th generation CPU requires 0.158 s; this is within the input image generation time, which is 0.2 s.

**Figure 9:**
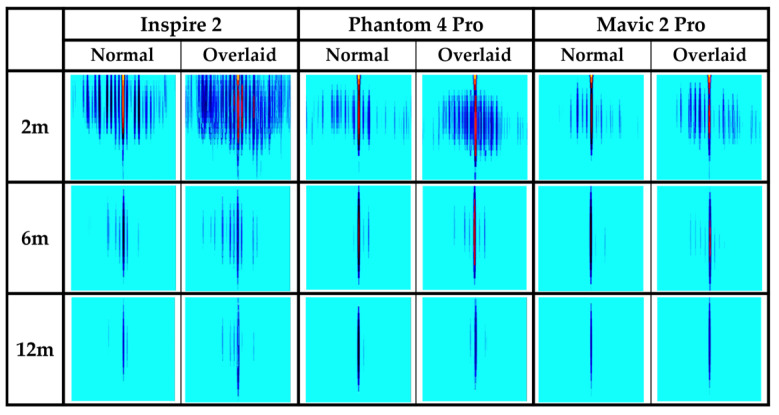
Normal images and overlaid images by distance and drone type.

Table 5 provides an overview of the datasets we created. Based on the optimization result for the number of overlaying images, we applied the image overlaying process detailed in Section 3.2, overlaying six range–Doppler map images to produce a single overlaid image. Subsequently, we generated 600 overlaid images from 3600 normal images for each drone model at every distance. Examples of normal and overlaid images for distances of 2, 6, and 12 m are depicted in Figure 9. It is clear from the images that the MDS is more prominent in the overlaid images compared to the normal ones.

## 5. Simulation Method and Results

This section confirms the efficacy of image overlaying in applying machine learning techniques to detect drones in different environments. Unfortunately, no open dataset is available for evaluating drone detection performance using range–Doppler map images. To resolve this issue, we compared the drone detection performance of the proposed image overlaying method. We compared it with conventional single-range–Doppler map-based machine learning methods. These conventional methods are detailed in references [10,11].

### 5.1. Drone Detection Accuracy Comparison

It is required to validate the effectiveness of the proposed overlaying method compared to the conventional drone detection methods applying a single range–Doppler map. As reference material for performance comparison, we want to evaluate the classification accuracy between drone and non-drone categories using a single-range–Doppler-map-image-based SVM [10] and CNN [11]; however, there are some problems with this performance comparison. There is no open FMCW range–Doppler map dataset for performance comparison, and the dataset used in the references is also unavailable; therefore, we compared the effect of image overlaying on drone detection performance by applying our range–Doppler map dataset, explained in Section 4.2. We created datasets composed of range–Doppler maps at various distances for experimentation. Table 6 summarizes the parameters of the SVM and CNN models used for the performance evaluation. The number of datasets was set similarly to [10], and we used the radial basis function (RBF) kernel type, as in [10]. We adopted the same method as [10], where the polynomial coefficient r was varied from 1 to 5, and the average drone detection accuracy was calculated.

In [11], the GoogLeNet model, one of the CNN architectures, was employed. We used the same model for our study and additionally conducted simulations using the AlexNet model, which has a simpler structure compared to GoogLeNet, to validate across multiple models. The number of datasets, epochs, learning rate, and batch size were set to be as similar as possible to those in [11]. The proportions of datasets were reasonably set, considering the number of datasets available.

In Table 7, we summarize the accuracy of detecting the presence of drones using conventional normal images and the proposed overlaid images with an SVM and CNN. For the SVM classifier, the accuracy of distinguishing drones from non-drones using normal images is 67.75%; however, the drone detection accuracy increased to 80.29% when using overlaid images, which is a significant improvement. Using the GoogLeNet model, a type of CNN, the accuracy of distinguishing drones from non-drones using normal images reached 96.72%. Similar to an SVM, the use of overlaid images led to an increase in accuracy, with an increment of 2.24%p, resulting in a detection accuracy of 99.96%. Additionally, when using the AlexNet model, there was 7.62%p increase in drone detection accuracy when using overlaid images. These findings indicate a clear improvement in detection accuracy when applying image overlay technology compared to using normal images.

### 5.2. The Performance of Drone Detection Results by Distance

In this subsection, we observed and analyzed the effectiveness of the proposed overlay method in determining the presence of drones categorized by drone models. We examined the presence of drones at various distances to observe how much the proposed image overlay technology contributes to increased drone detection range. 

The training set was created based on distance categories and divided into “Drone” and “No Drone” categories. The “Drone” category included data from Inspire 2, Phantom 4 Pro, and Mavic 2 Pro at various distances, while the “No Drone” category contained data from non-drone objects. Drone detection accuracy was calculated using test set images that were not used for training.

As shown in Table 7, we utilized different CNN models, such as AlexNet, SqueezeNet, and GoogLeNet. Each model exhibits varying levels of complexity, with AlexNet being the simplest, followed by SqueezeNet and then GoogLeNet, which is the most structurally complex. Despite being trained on the same dataset, these models capture different features and nuances, thereby enabling more comprehensive learning. By leveraging a diverse range of models, we facilitate generalized learning, allowing the network to adapt to various scenarios and enhance its overall performance effectively.

As outlined in Table 5, consisting of a specific number of samples, we structured the dataset split into training, validation, and test sets based on the proportions of 70%, 15%, and 15%, respectively. Notably, since the “Drone” category encompasses data from three different drone models, the quantity of “No Drone” data is three times less. To address this class imbalance, we attempted data augmentation specifically for the “No Drone” class by randomly adjusting image sizes within 10% margins.

**Figure 10:**
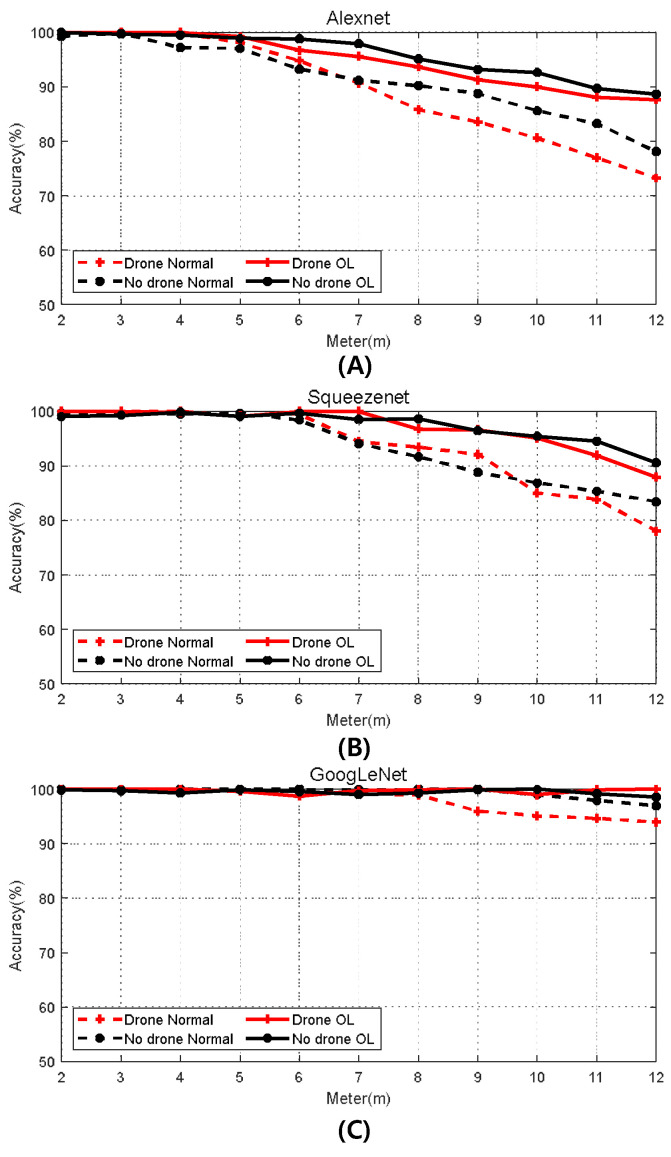
Drone classification accuracy via the deep learning model. ((**A**): Alexnet, (**B**): Squeezenet, (**C**): GoogLeNet).

Figure 10A–C show graphs representing the accuracy of drone detection using AlexNet, SqueezeNet, and GoogLeNet, respectively. The “Drone” category is depicted in red, while the “No Drone” category is represented in black. Solid lines show the detection accuracy of normal images, while dotted lines show the detection accuracy of overlaid images for comparison. As indicated in Figure 9, as the distance between the drone and the radar increases, the MDS weakens, making it more difficult to distinguish between the “Drone” and “No Drone” categories. Analyzing the graphs, it is evident that at distances of 3 m or less, both the normal and overlaid images of the “No Drone” data exhibit detection accuracies close to 100%.

Upon analyzing the results using the AlexNet model, it is observed that beyond 3 m, the accuracy of the normal images decreases steeply, leading to a drop in drone detection accuracy to below 80%. Similar trends in decreasing drone detection accuracy with increasing distance are observed with SqueezeNet and GoogLeNet; however, for the overlaid images, the MDS of the drone remains distinct, resulting in a drone detection accuracy of around 90%, even at the furthest distance of 12 m. Notably, the drone detection accuracy of overlaid images surpasses that of normal images at all distances, indicating the effectiveness of the overlay technique in enhancing drone detection, particularly at longer distances.

### 5.3. The Performance of Drone Detection Results When Detecting an Unknown Drone

To validate the efficiency of the proposed method, we examined whether untrained drone images could be classified into the drone category when input into a trained network with other drone models. The training dataset included images of all drones except the ones designated for detection in the “Drone” category, as shown in Table 8. The SqueezeNet network was used for the machine learning model.

**Figure 11:**
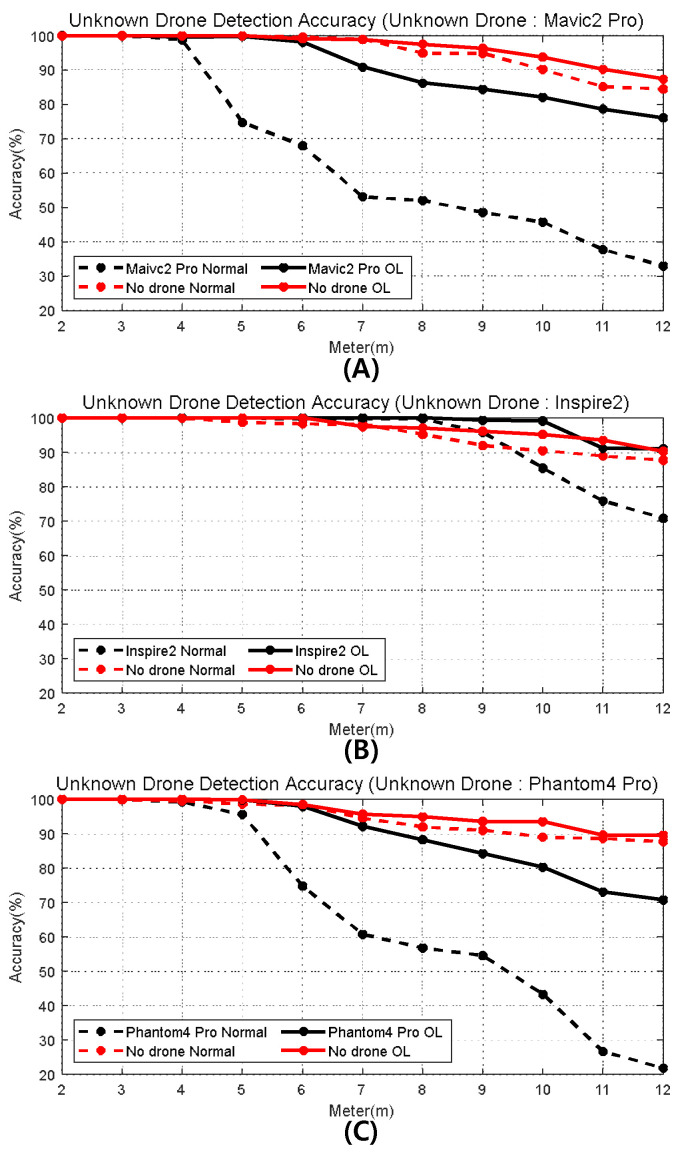
Unknown drone detection accuracy ((**A**): Case1, (**B**): Case2, and (**C**): Case3).

Figure 11 illustrates the accuracy of the drone detection and false alarms of untrained drone scenarios in Table 8. For all three scenarios, the detection and false alarm performance with the overlaid images are noticeably better than those with the normal images. Based on the 80% detection probability, when using the normal image, the distances are only within 4.8, 10.5, and 5.8 m for Mavic 2 Pro, Inspire 2, and Phantom 4 Pro, respectively; however, when using the overlaid image, the distances are increased to 10.5, more than 12, and 10.0 m, respectively. Based on the 10% false alarm probability, when using the normal images, the acceptable distances are only within 10, 10, and 9.5 m for Mavic 2 Pro, Inspire 2, and Phantom 4 Pro, respectively. Still, when using the overlaid image, the distances are 11.0, 12.0, and 12.0 m, respectively, an average increase of 1.8 m.

The results demonstrate that using the proposed image overlay technology directly correlates with an increase in drone detection distance. Specifically, when examining the unknown drone detection accuracy when the Mavic 2 Pro is the unknown drone, for the 80% detection probability, the detection range is extended from 4.8 m to 10.5 m. Although a difference of 5.7 m in the detection range might seem minimal, it is more than a 119%p increment.

The reason for our study’s limited observation range of 12 m is the limitation of the FMCW radar that we used. It operates in the 24 GHz ISM band with limited transmission power. If the proposed overlay method is applied to commercial FMCW radars with higher transmission power, like the LR500 in Korea [20], which operates in the X-band with a transmission power of 53 dBm, and the “UAVX” system from BlackSage Technology Boise, ID, USA [21], which uses an X-band radar with 40 dBm, it would be possible to detect objects at much greater distances. Consequently, the image overlay technique would be more effective, enhancing detection capabilities over extended ranges. For example, if the detection range of Mavic 2 Pro for the conventional commercial radar with the normal range–Doppler image is 500 m, it could be extended to 1059 m.

## 6. Conclusions

We propose an “image overlay technique” to improve the detection accuracy of drones at longer distances. This technique involves overlaying range–Doppler images to sharpen the MDS. When we compared the drone detection accuracy results, the proposed image overlaying method significantly outperformed drone detection with normal single range–Doppler images, particularly for drones at longer distances. Our study is limited to using image processing techniques to combine the MDSs. However, we expect further improvements in performance if the MDS signals from different time slots are coherently combined in a more optimized manner. It could be a promising topic for future studies. In a future study, we aim to integrate information from multiple sensors, e.g., FMCW radar, CW radar, and radio frequency (RF) receivers, to determine the presence of drones and distinguish their models.

## Figures and Tables

**Table 1:** Brief summary of the artwork using an MDS.

References	Feature	Target
[3]	TVD	Drone, bird, noise
[6,7]	TVD	Drone
[10,11]	Range–Doppler Map	Drone, etc.
[9]	Range–Doppler Map	Drone

**Table 2:** Specifications of drones used in the experiment.

	Inspire 2	Phantom 4 Pro	Mavic 2 Pro
Size (cm)	L: 42.7	L: 28.95	L: 32.2
	H: 31.7	H: 28.95	H: 24.2
	W: 42.5	W: 19.6	W: 8.4
Propeller. size (cm)	38	24	22

**Table 3:** Operating parameters of FMCW radars.

Name	Eval-DEMORAD (Analog Device)
Frequency	24 GHz (K band)
Start–stop frequency	24.00–24.25 GHz
Number of chirps	128
Upchirp duration	256 μs
Sampling frequency	1 MHz
Number of samples for one chirp	256
Transmit power	8 dBm

**Table 4:** Machine learning parameters for a drone classification experiment according to the number of overlays.

Parameter	CNN (AlexNet)	
Image size (pixel)	227 × 227 × 3	
Number of overlaid images	Drone	No drone
	1000	1000
Dataset ratio training/validation/test	70%/15%/15%	
Learning rate	0.001	
Batch size	64	

**Table 5:** Overall datasets for the range–Doppler maps of targets.

Data Existence by Distance (2~12 m)	Dataset Construction	
	Normal Image	Overlaid Image
Inspire 2	3600	600
Mavic 2 Pro	3600	600
Phantom 4 Pro	3600	600
No drone	3600	600

**Table 6:** Parameter for machine learning.

Parameters	SVM			CNN		
Image size (pixel)	224 × 224 × 3			224 × 224 × 3 (227 × 227 × 3)		
Num. of images	Normal	Drone	6000	Normal	Drone	13,200
		No drone	6000		No drone	13,200
	OL	Drone	1000	OL	Drone	2200
		No drone	1000		No drone	2200
Dataset ratio training/validation/test	70%/30% (no validation in SVM)			70%/15%/15%		
Kernal	RBF			-		
Iteration	500			-		
Epoch	-			40		
Learning rate	0.001			0.001		
Batch size	50			64		

**Table 7:** Comparison of drone detection methods and their accuracy.

Method		Accuracy
SVM	[10]	67.75%
	Proposed	80.29%
GoogLeNet	[10]	96.72%
	Proposed	99.96%
AlexNet	[10]	87.69%
	Proposed	95.31%

**Table 8:** Category classification of Drone and No Drone by case.

	Training Data		Test Data
Category	Drone Category	No Drone Category	Unknown Drone
Case1	Inspire 2, Phantom 4 Pro	No drone	Mavic 2 Pro
Case2	Phantom 4 Pro, Mavic 2 Pro	No drone	Inspire 2
Case3	Inspire 2, Mavic 2 Pro	No drone	Phantom 4 Pro

## Data Availability

The data presented in this study are available upon request from the corresponding author with restrictions. The data are not publicly available due to the policy of the institute.
